# SLC2A8 (GLUT8) is a mammalian trehalose transporter required for trehalose-induced autophagy

**DOI:** 10.1038/srep38586

**Published:** 2016-12-06

**Authors:** Allyson L. Mayer, Cassandra B. Higgins, Monique R. Heitmeier, Thomas E. Kraft, Xia Qian, Jan R. Crowley, Krzysztof L. Hyrc, Wandy L. Beatty, Kevin E. Yarasheski, Paul W. Hruz, Brian J. DeBosch

**Affiliations:** 1 Department of Pediatrics, Washington University School of Medicine, 660 S. Euclid Ave., St. Louis, MO 63110, USA; 2 Department of Medicine, Washington University School of Medicine, 660 S. Euclid Ave., St. Louis, MO 63110, USA; 3 Center for the Investigation of Membrane Excitability Diseases, Washington University School of Medicine, 660 S. Euclid Ave., St. Louis, MO 63110, USA; 4 The Hope Center for Neurological Disorders, Alafi Neuroimaging Laboratory, Washington University School of Medicine, 660 S. Euclid Ave., St. Louis, MO 63110, USA; 5 Department of Molecular Microbiology, Washington University School of Medicine, 660 S. Euclid Ave., St. Louis, MO 63110, USA; 6 Department of Cell Biology & Physiology, Washington University School of Medicine, 660 S. Euclid Ave., St. Louis, MO 63110, USA

## Abstract

Trehalose is a disaccharide demonstrated to mitigate disease burden in multiple murine neurodegenerative models. We recently revealed that trehalose rapidly induces hepatic autophagy and abrogates hepatic steatosis by inhibiting hexose transport via the SLC2A family of facilitative transporters. Prior studies, however, postulate that intracellular trehalose is sufficient to induce cellular autophagy. The objective of the current study was to identify the means by which trehalose accesses the hepatocyte cytoplasm, and define the distal signaling mechanisms by which trehalose induces autophagy. We provide gas chromatographic/mass spectrometric, fluorescence microscopic and radiolabeled uptake evidence that trehalose traverses the plasma membrane via SLC2A8 (GLUT8), a homolog of the trehalose transporter-1 (Tret1). Moreover, GLUT8-deficient hepatocytes and GLUT8-deficient mice exposed to trehalose resisted trehalose-induced AMP-activated protein kinase (AMPK) phosphorylation and autophagic induction *in vitro* and *in vivo*. Although trehalose profoundly attenuated mTORC1 signaling, trehalose-induced mTORC1 suppression was insufficient to activate autophagy in the absence of AMPK or GLUT8. Strikingly, transient, heterologous Tret1 overexpression reconstituted autophagic flux and AMPK signaling defects in GLUT8-deficient hepatocyte cultures. Together, these data suggest that cytoplasmic trehalose access is carrier-mediated, and that GLUT8 is a mammalian trehalose transporter required for hepatocyte trehalose-induced autophagy and signal transduction.

The role of cellular macroautophagy (hereafter, “autophagy”) in manifold disease processes is a target of intense investigation. Autophagy is a tightly regulated process that ensues as the end-manifestation of cellular stressors such as starvation, protein and fat accumulation^1^,^2^.

Trehalose is a disaccharide previously demonstrated to induce autophagy and mitigate multiple disease models through incompletely defined mechanisms^3^,^4^,^5^,^6^,^7^,^8^,^9^. This body of work established trehalose as an attractive nutraceutical to interrogate mechanistically, and indeed, the prospect that trehalose – or a ‘designer drug’ based upon the trehalose mechanism of action – will translate into a *bona fide* human therapeutic for metabolic disease is gaining traction^10^.

The glucose transporter (GLUT, encoded by *slc2a* genes) family of transmembrane-spanning facilitative carbohydrate transporters are ubiquitously expressed carriers that mediate transport of carbohydrates and other substrates down their concentration gradients across lipid bilayers^11^. These carriers thus dictate cellular energetics and glucose sensing at the cellular, organ and organism levels^11^,^12^,^13^,^14^,^15^,^16^,^17^,^18^. We recently showed that trehalose inhibits the GLUT family of carbohydrate carrier homologs to induce hepatic autophagic flux and protection from hepatic steatosis in an ATG16L1- and AMPK-dependent manner^10^,^19^. Prior work, however, indicated that intracellular trehalose is sufficient for trehalose-induced autophagy^3^,^10^. The rapid kinetics of AMPK activation and autophagic induction, combined with the putative intracellular space in which trehalose exerts these actions necessitated a rapid means by which trehalose could access the hepatocyte interior. Yet, despite identification of facilitative trehalose transport in lower organisms^20^, the means by which a disaccharide could efficiently access the interior of a mammalian cell is heretofore unknown.

Here, our objective was to resolve the signaling events mediating trehalose-induced autophagy and the mechanisms by which trehalose induces these signaling events. We provide evidence that trehalose accesses the hepatocyte cytoplasm, in part, via GLUT8, the mammalian homolog of the trehalose transporter, Tret1. We further provide evidence that GLUT8 is essential for trehalose-induced activation of the AMPK-ULK1 pathway and autophagic flux in a way that is genetically complemented by heterologous Tret1 expression. We then demonstrate that trehalose suppresses hepatic mTORC1 signaling in a GLUT8-independent fashion, and that trehalose-mediated mTORC1 suppression is strikingly insufficient to induce hepatic autophagy in the absence of GLUT8 and AMPK. Together, our data unify prior data regarding the mechanism of action of trehalose, and support GLUT8-dependent and GLUT8-independent functions in the acute actions of trehalose.

## Results

### GLUT8 is a trehalose transporter homolog

**Figure 1:**
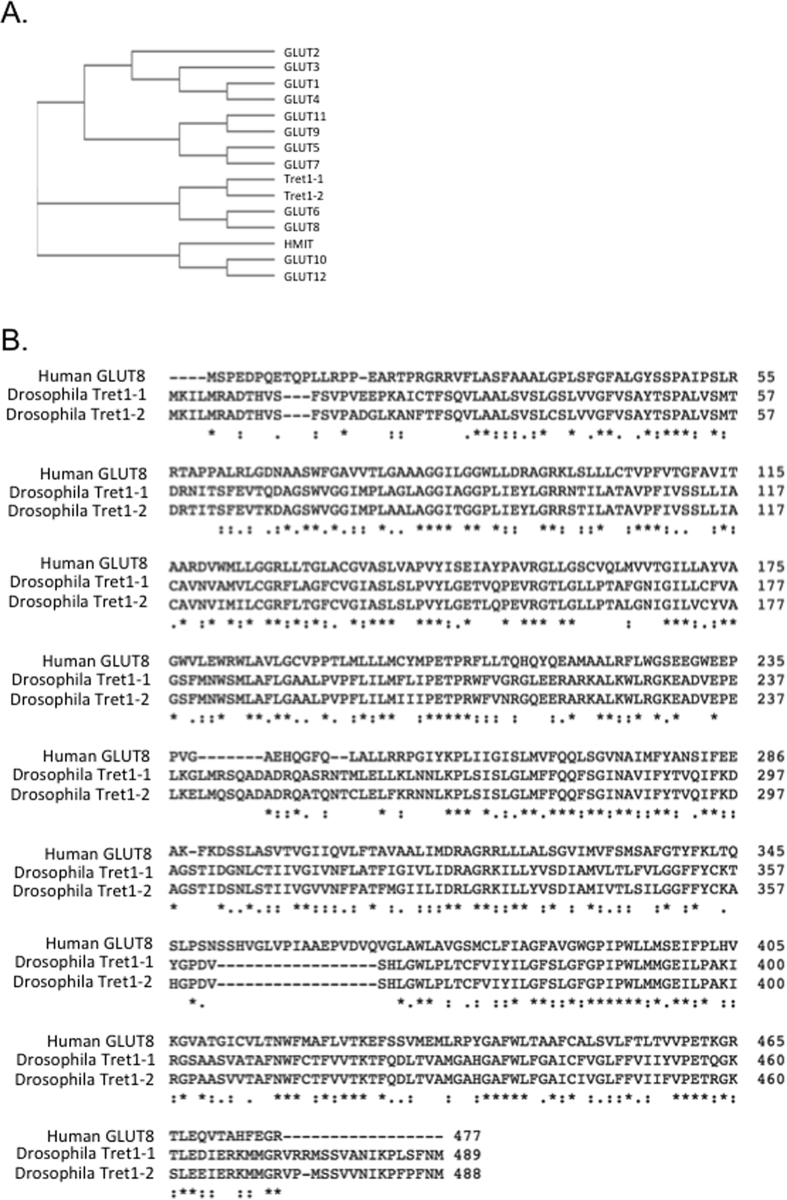
SLC2A8 is a trehalose transporter homolog. (**A**) ClustalΩ multiple sequence alignment demonstrating homology of human GLUT family members with high-capacity trehalose transporters from drosophila melanogaster, Tret1-1 and Tret1-2. (**B**) Multiple pairwise alignment demonstrating amino acid sequence homology between human GLUT8, Tret1-1 and Tret1-2. Lines denote amino acid identity; paired dots denote conservative sequence and single dots indicate semi-conservative residues.

We demonstrated that SLC2A8 aligns closely with specialized trehalose-binding enzymes – murine trehalase and the drosophila trehalose receptor, Tre1^19^. We tested whether the primary amino acid sequences of human GLUT8 and its closely related class III GLUT homologs also significantly aligned with specialized trehalose transporter proteins, drosophila Tret1-1 and Tret1-2, using ClustalΩ (Fig. 1A). GLUT6 and GLUT8 – and to a lesser extent, GLUT 10, 12 and HMIT – shared common evolutionary branches with dTret1-1 and dTret1-2. In contrast, the class I and class II GLUTs (e.g. GLUT1-4, GLUT5, 7, 9, 11) exhibited more divergent primary structures. To quantify the extent of homology between GLUT8, Tret1-1 and Tret1-2 trehalose transporters, we conducted simultaneous alignment using ClustalΩ (Fig. 1B). This demonstrated near identity between Tret1 variants along the entire length of GLUT8, suggesting that GLUT8 might harbor specialized trehalose transporter properties similar to that of Tret1.

### Trehalose is rapidly transported into hepatocytes in a GLUT8-dependent manner

**Figure 2:**
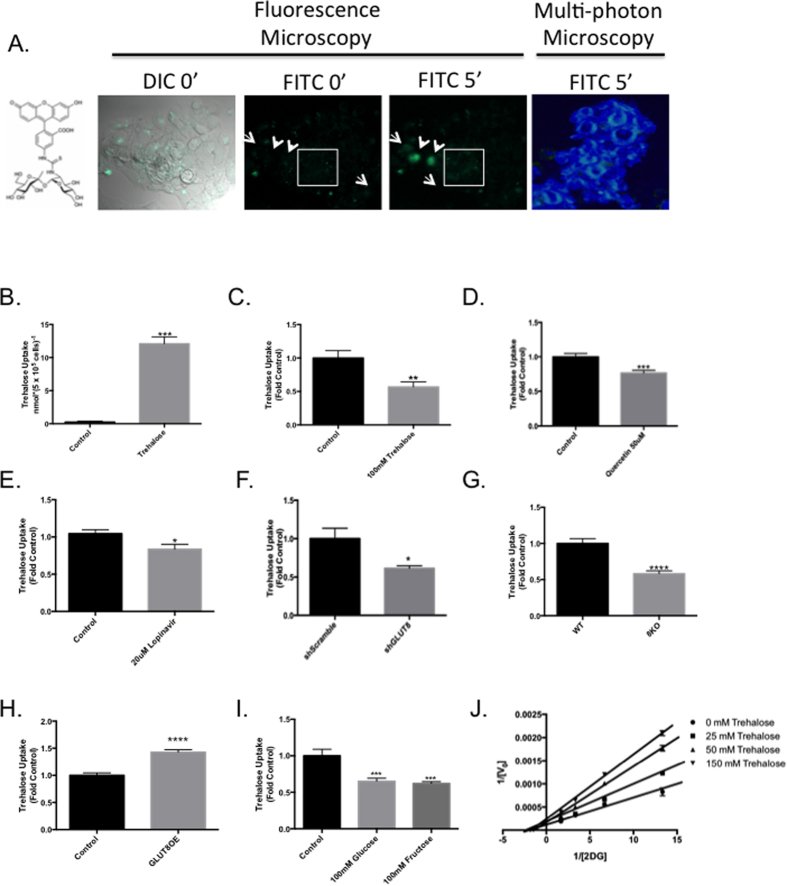
GLUT8 is a mammalian trehalose transporter. (**A**) Standard fluorescence microscopy and multi-photon microscopy demonstrating HepG2 fluorescein isothiocyanate (FITC)-labeled trehalose uptake. Left panel, chemical structure of probe demonstrating covalent FITC conjugation of trehalose. Left to right, DIC and green-fluorescent channel views at 0′ and 5′ post-FITC pulse labeling. Green fluorescence represents FITC fluorescence. 0′-treated (no FITC label added) cultures are shown to demonstrate autofluorescence. Right panel, Multi-photon microscopic analysis of FITC label uptake. Blue coloring represents cellular autofluorescence; green fluorescence represents FITC label (**B**). Gas chromatography-mass spectrometric analysis of HepG2 hepatocytes incubated in the presence or absence of 100 mM trehalose (5 minutes) (**C**). Five-minute [^14^C]-trehalose uptake in wild-type primary murine hepatocyte cultures in the presence or absence of 100 mM unlabeled trehalose. (**D**) [^14^C]-trehalose uptake in primary murine hepatocyte cultures preincubated in the presence or absence of the flavone glucose transporter inhibitor, quercetin (50 μM). (**E**) [^14^C]-trehalose uptake in primary murine hepatocytes pre-incubated with DMSO (Control) or the GLUT8-selective inhibitor lopinavir (20 μM). (**F**) [^14^C]-trehalose uptake in HepG2 hepatoblastoma cell lines stably expressing scrambled (shScramble) or GLUT8-specific (shGLUT8) shRNA. (**G**) [^14^C]-trehalose uptake in primary murine hepatocytes derived from wild-type (WT) or germline GLUT8-deficient (8KO) mice. (**H**) [^14^C]-trehalose uptake (180 minutes) measured in 293 cell lines overexpressing vector alone (Control) or hGLUT8 (GLUT8OE). (**I**) Five-minute [^14^C]-trehalose uptake measured in GLUT8OE cell lines pre-treated with 100 mM unlabeled glucose or fructose. (**J**) Lineweaver-Burke plot of GLUT8OE cell trehalose uptake (5 minute uptake period) in the presence of varying concentrations of radiolabeled 2DG and varying concentrations of trehalose. *, **, *** and ****P < 0.05, P < 0.01, P < 0.001 and P < 0.0001, respectively versus control by 2-tail homoscedastic T-test with Bonferroni-Dunn post hoc correction for multiple comparisons.

Substantial GLUT8 homology to specialized trehalose transporters prompted us to test, using mutiple complementary methodologies, whether trehalose is transported into hepatocytes. We first asked whether trehalose accesses the cytoplasm under direct microscopic visualization. WT primary murine hepatocytes were hexose-starved in glucose-free HEPES, then incubated (37 °C, 5 minutes) with FITC-labeled trehalose. Standard immunofluorescence microscopy further demonstrated minimal fluorescence preceding labeling, with scattered trehalose uptake of variegated intensity 5-minutes post-FITC labeling (Fig. 2A).

We next quantified intact disaccharide accumulation directly using gas chromatography-mass spectroscopic analysis. HepG2 cultures were incubated with or without 100 mM trehalose (5 minutes) prior to extensive washing, lysis and GC-MS analysis of soluble methanol extracts. An uptake rate of 10–12 nmol trehalose*5 × 10^5^ cells^−1^*min^−1^ was observed in trehalose-treated cultures (P < 0.001 versus untreated cultures), suggesting intact trehalose accumulation within the hepatocyte in the absence of mass changes, (e.g. by phosphorylation or cleavage, Fig. 2B). These uptake rate data are comparable to HPLC-based trehalose uptake data obtained in Huh-7 hepatoma, NIH/3T3–3–4 and CHO-K1 cell lines overexpressing Tret1 from *P Vanderplankii*^20^.

Given the data suggesting that trehalose accumulates in the cytoplasm, we tested whether trehalose uptake is carrier-mediated. This was first explored by determining whether [^14^C]-trehalose uptake is competitively inhibited by unlabeled trehalose (Fig. 2C). Primary mouse hepatocytes were incubated in glucose-free HEPES in the presence or absence of 100 mM unlabeled trehalose, after which cultures were pulsed (37 °C, 5 minutes,) with [^14^C]-trehalose with or without unlabeled trehalose in the uptake media. 100 mM unlabeled trehalose inhibited radiolabeled trehalose uptake by 50% relative to control cultures (Fig. 2C).

We then determined whether GLUT-selective inhibition modulates radiolabeled trehalose uptake. Primary hepatocytes were glucose-starved in the presence of DMSO or of the non-selective flavonoid GLUT inhibitor, quercetin (50 μM)^21^,^22^ 15 minutes prior to radiolabeled trehalose uptake measurements. Quercetin significantly reduced trehalose uptake by approximately 25% (Fig. 2D, P < 0.001) versus control cultures. Similarly, we tested whether the isoform-selective GLUT inhibitor lopinavir^23^ blocked radiolabeled trehalose uptake by incubating primary hepatocyte cultures with DMSO (“control”) or with 20 μM lopinavir during 15′ glucose starvation prior to radiolabeled trehalose uptake assay. Lopinavir inhibited trehalose uptake again by approximately 25% relative to DMSO-treated cultures at 5 minutes of radiolabel incubation (Fig. 2E).

GLUT2 and GLUT8 are the most abundant hexose transporters in mouse and human liver^17^. In light of significant homology between GLUT8 and Tret1, we examined whether GLUT8 mediates hepatocyte trehalose transport by measuring [^14^C]-trehalose uptake in glucose-starved HepG2 cells stably expressing scrambled (“control”) shRNA or GLUT8-directed (“GLUT8′) shRNA. qRT-PCR analysis of GLUT isoforms 1–12 revealed that shGLUT8 cells exhibit 85% knockdown of GLUT8 mRNA relative to control cultures without significant alterations in any other GLUT (Supplementary Fig. 1A). Moreover, GLUT8 protein abundance was significantly reduced in these cells (~60%) relative to control cells (Supplementary Fig. 1B). Accordingly, one-minute uptake of [^3^H]-2-deoxyglucose (2DG) in shGLUT8 cells was attenuated by 76% relative to shScramble controls (Supplementary Fig. 1C and as previously reported^17^). Consistent with these findings, GLUT8-deficient cultures exhibited ~40% lower trehalose uptake relative to scrambled shRNA-expressing control cultures (Fig. 2F). Similarly, we analyzed radiolabeled trehalose uptake in glucose-starved primary murine hepatocytes derived from wild-type (WT) or GLUT8KO mice. GLUT8KO primary hepatocytes exhibited 42% lower trehalose uptake at 5 minutes (Fig. 2G).

The above data suggested that GLUT8 is required for maximal hepatocyte trehalose uptake. We therefore determined whether GLUT8 expression is sufficient to mediate trehalose uptake in a 293 GLUT8 overexpression system, which was previously utilized to study single-transporter biology in mammalian cells^19^,^23^ due to knockdown of endogenous GLUT1. 293 cells incubated in glucose-free HEPES were then pulsed with radiolabeled trehalose. In agreement with experiments in Tret1-overexpressing cell lines^20^, human GLUT8 overexpression significantly increased trehalose uptake in 293 cell cultures when compared with cultures expressing empty vector (Fig. 2H). Moreover, trehalose uptake in this GLUT8-enriched transport model system was competitively inhibited by known substrates of GLUT8^11^,^15^,^24^,^25^ - including glucose and fructose (each 100 mM, Fig. 2I).

Recent work demonstrated that trehalose inhibits glucose uptake mediated through SLC2A proteins^19^. We therefore analyzed whether enzyme kinetic data were consistent with competitive, mixed, or non-competitive inhibition. HEK293 cultures were incubated in the presence of increasing concentrations of trehalose and radiolabeled 2-deoxy-D-glucose (2DG) prior to assaying 2DG uptake. Consistent with binding and inhibition to either the inward open transporter conformation or with binding off the active site, Lineweaver-Burke analysis revealed that trehalose increased K_m_/V_max_ ratio for outside-in 2DG uptake in a dose-dependent manner, (Fig. 2J), concomitant with dose-dependent decreases in apparent V_max_.

### Binding of trehalose in the glucose-binding pocket of GLUT8

**Figure 3:**
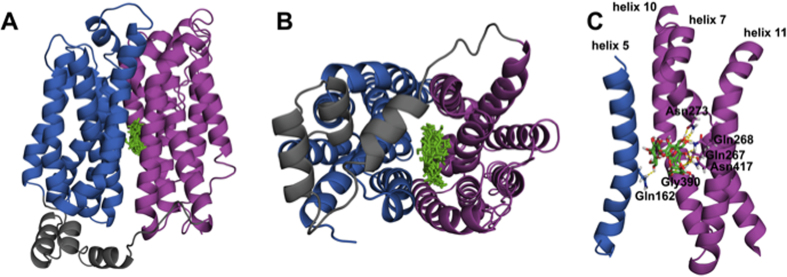
Trehalose docks in the inward open glucose binding pore of GLUT8. (**A**) Lowest-energy interaction model between GLUT8 (ribbons) and trehalose (in green). Grey ribbons demarcate intracellular domains. Red and blue ribbons denote transmembrane domains. (**B**) 90° Z-plane rotation looking down the carbohydrate binding pore of the model shown in (**A**). (**C**) Putative residues within the Glucose binding pore that mediate the trehalose-transporter interaction.

In order to further investigate the potential interactions between trehalose and GLUT8, we perfomed *in silico* docking analysis using modeled structures of human GLUT8. As the crystallographic structure of GLUT8 is not yet reported, we modeled its structure using the I-TASSER modeling server with the open, inward facing crystal structures of human GLUT1, bovine GLUT5 and bacterial XylE as templates. Trehalose was docked to three distinct GLUT8 structures, each originating from a different template. Using rigid receptor, flexible ligand docking, we selected the entire GLUT8 structure as docking space. >80% of trehalose poses bind at a central location within the glucose permeation pathway of GLUT8 (Fig. 3A,B). Closer examination of the amino acids involved in binding trehalose revealed the highly conserved residues Gln162, Gln267, Gln268, Asn273, Gly390 and Asn417 to form polar interactions between the ligand and GLUT8 in all three examined models (Fig. 3C). With the exception of Gly390, all of these residues are involved in binding glucose in the human homologue GLUT1 and constitute >80% of the hydrogen bond network at the glucose binding site^26^. These results indicate that trehalose binding at the monosaccharide binding pore is energetically favorable over other transporter sites in the inward open transporter conformation.

### Trehalose induces ULK1-dependent autophagy independent of AMPK-mTORC1 crosstalk

**Figure 4:**
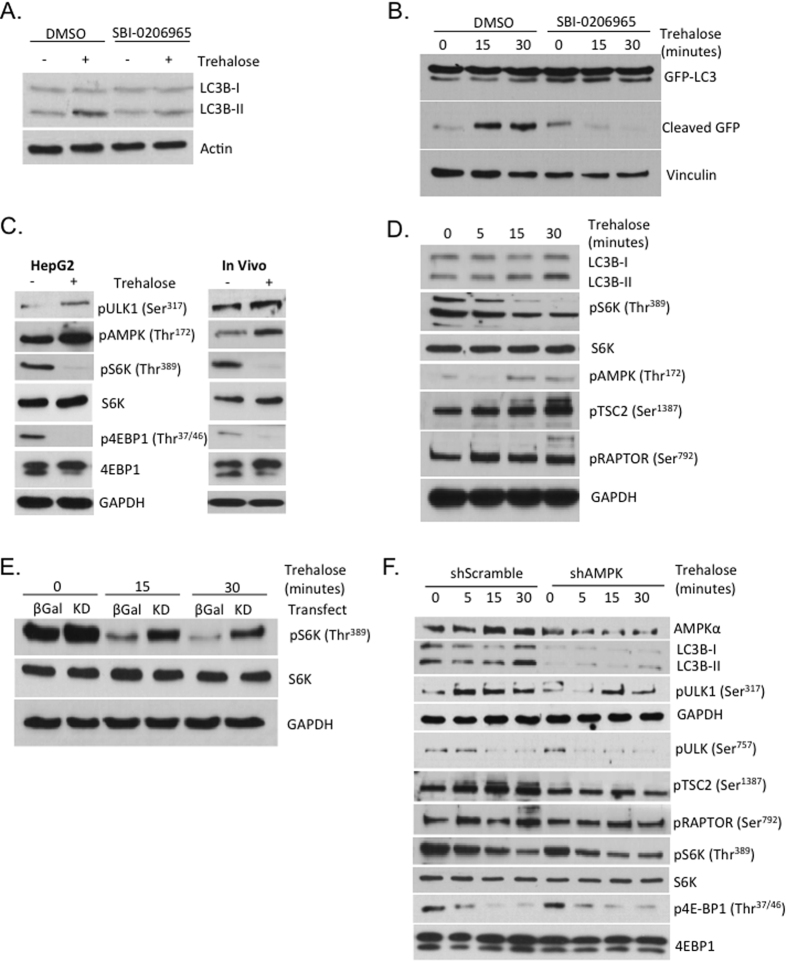
AMPK and ULK1 activity – but not mTORC1 suppression - mediate trehalose-induced autophagy. (**A**) ULK1 is required for maximal trehalose-induced LC3B-II accumulation. HepG2 cells were pre-treated with DMSO or with the ULK1-specific inhibitor, SBI-0206965 for 30′ prior to stimulation with trehalose (100 mm) for 0 – 30′. GAPDH and LC3B immunoreactivity were quantified in whole-cell lysates. (**B**) ULK1 is required for trehalose-induced GFP-LC3 cleavage. Primary murine hepatocyte derived from mice overexpressing GFP-LC3 fusion protein were stimulated with 100 mM trehalose for 0-30′ following pre-incubation with DMSO or with SBI-0206965. GFP-specific immunoblotting was performed in whole-cell lysates. Shown are total immunoreactive GFP-LC3 (upper panel), cleaved GFP (middle panel) and vinculin as a loading control. (**C**) LC3B-II accumulation and ULK1 (Ser^317^) and AMPK (Thr^172^) phosphorylation are associated with decreased S6 kinase (Thr^389^) and 4E-BP1 (Thr^37/46^) phosphorylation. Shown are phospho-specific immunoblots in HepG2 cells (left panels) treated with or without 100 mM trehalose (30 min.) or in liver lysates from GFP-LC3^WT^ mice 30 minutes after oral (6 g/kg) trehalose gavage. (**D**) Trehalose induces AMPK and mTORC1 cross-talk via TSC2 (Ser^1387^) and raptor (Ser^792^) phosphorylation. LC3B and phospho-specific target immunoblotting is demonstrated in whole-cell lysates derived from HepG2 cultures treated with growth media (depicted as 0′) or with 100 mM trehalose for up to 30 minutes. GAPDH is demonstrated as a loading control (bottom panel). (**E**) Kinase-dead AMPK attenuates trehalose suppression of mTORC1 signaling. HepG2 cultures were transfected with β-galactosidase (β Gal) or kinase-dead AMPK (KD) and treated with or without 100 mM trehalose for 30′. Phospho-S6K and GAPDH bands are shown in upper and lower panels, respectively. (**F**) Trehalose-induced mTORC1 blockade is insufficient for trehalose-induced autophagy. HepG2 cultures transfected with scrambled or AMPK-directed shRNA were stimulated with or without 100 mM trehalose (0-30 minutes). Lysates were immunoblotted for total AMPKα, LC3B, GAPDH, or for phospho-specific AMPK targets - ULK1 (Ser^317^), TSC2 (Ser^1387^), Raptor (Ser^792^), and mTORC1 targets – ULK1 (Ser^757^) S6 kinase (Thr^389^) and 4E-BP1 (Thr^37/43^).

Prior work demonstrated that trehalose activates AMPK (Thr^172^) phosphorylation, which in turn activates ULK1 via phosphorylation at the AMP-activated protein kinase (AMPK) substrate site, Ser^317^ within 30 minutes post-trehalose stimulation *in vitro* and *in vivo*^19^. This was associated with decreased ULK1 phosphorylation at the putative “inhibitory” site phosphorylated by mTORC1, ULK1 (Ser^757^)^27^. Because both AMPK activation and mTORC1 inhibition converge at ULK1 to stimulate autophagic flux^27^, we first tested directly whether ULK1 is required for trehalose-induced autophagic flux. HepG2 cultures were incubated (30 minutes) in regular growth media containing DMSO or SBI-0206965, a competitive ULK1-specific kinase inhibitor^28^, prior to stimulation with trehalose (100 mM) for 0–30 minutes. Immunoblot analysis of whole-cell lysates revealed enhanced LC3B-II accumulation in DMSO-treated cells stimulated with trehalose (Fig. 4A). Additionally, trehalose stimulated p62 degradation, but over a protracted timecourse (Supplementary Fig. 2) in wild-type HepG2 and primary hepatocytes. Regardless, the rapid LC3B-II accumulation response was attenuated in HepG2 cultures treated with SBI-0206965 (Fig. 4A. Densitometric quantification data for Fig. 4 are catalogued in Supplemental Fig. 3).

To test the role of ULK1 in trehalose-stimulated autophagy in a primary hepatocyte culture system, primary murine hepatocytes were isolated from mice constitutively expressing a transgene encoding a GFP-LC3 fusion protein (GFP-LC3^WT^), from which GFP is cleaved upon autophagic activation^29^,^30^. DMSO-treated hepatocytes stimulated with trehalose rapidly induced GFP cleavage within 30 minutes. In contrast, hepatocytes treated with SBI-0206965 exhibited an attenuated GFP cleavage response to trehalose over the same timecourse (Fig. 4B).

To examine coordinated trehalose-induced mTORC1 and AMPK regulation upstream of ULK1, HepG2 cells were incubated in growth media or in growth media containing 100 mM trehalose (30 minutes). Lysis and immunoblot analysis following trehalose stimulation demonstrated enhanced AMPK (Thr^172^) and ULK1 (Ser^317^) phosphorylation and LC3B-II accumulation, concomitant with attenuated phosphorylation of mTORC1 targets, ribosomal S6 protein kinase (S6K phospho-Thr^389^) and 4E-binding protein-1 (4E-BP1, phospho-Thr^37/46^) (Fig. 4C, left panels). *In vivo*, wild-type C57/B6 male mice were subjected to orogastric gavage (6 g/kg, 30 minutes), followed by liver dissection and immunoblotting for mTORC1 targets (Fig. 4C, right panels). Similar to findings in HepG2 cultures, immunoblot analysis of murine liver crude lysates revealed enhanced phosphorylated AMPK and ULK1 (Thr^172^ and Ser^317^, respectively) concomitant with decreased S6K (Thr^389^) and 4E-BP1 (Thr^37/46^) phosphorylation (Fig. 4C, right panels).

The above data suggested that ULK1 is required for trehalose-induced autophagic flux. Observations that AMPK and mTORC1 pathways were coordinately modulated by trehalose activation prompted us to assess whether AMPK→ mTORC1 crosstalk occurred at signaling nodes other than ULK1 (Ser^757^), including at TSC2 (Ser^1387^ 31) and at raptor (Ser^792^ 32). HepG2 cells were treated with regular growth media or with 100 mM trehalose for 5–30 minutes prior to immunoblot analysis of whole-cell lysates. Concomitant with LC3B-II accumulation, increased AMPK (Thr^172^) phosphorylation, and decreased S6K (Thr^389^) phosphorylation during the trehalose stimulation timecourse (Fig. 4D), enhanced TSC2 (Ser^1387^) and raptor (Ser^792^) phosphorylation at AMPK crosstalk-specific nodes within the mTORC1 pathway were observed in response to trehalose.

We demonstrated previously that AMPK inhibition using a dominant-negative, kinase-dead AMPKα (catalytic subunit) mutant was sufficient to prevent trehalose-induced autophagic flux^19^. To evaluate whether AMPK activity is required for trehalose to inhibit mTORC1 signaling, HepG2 cultures were transfected with β-galactosidase (β-gal) or with kinase-dead (KD) AMPK incubated in the presence or absence of 100 mM trehalose for 15 minutes. Basal phosphorylation of S6K (Thr^389^) was enhanced in KD-transfected cell cultures, and S6K (Thr^389^) phosphorylation remained relatively elevated throughout the 30-minute timecourse post-trehalose stimulation. Nevertheless, trehalose reduced mTORC1 signaling through S6K (Fig. 4E).

We next determined whether AMPK is required for trehalose-induced mTORC suppression in association with trehalose-induced autophagic induction. HepG2 cells were transiently transfected with AMPKα-directed shRNA-encoding (shAMPK) virus, or with scrambled shRNA-encoding adenovirus (shScramble) prior to 100 mM trehalose stimulation in regular growth media. LC3B-II increased during the 30-minute trehalose stimulation timecourse in shScramble cells. This was associated with enhanced AMPK signal transduction (e.g increased ULK1 (Ser^317^), TSC2 (Ser^1387^) and raptor (Ser^792^) phosphorylation), and with diminished mTORC1 signaling (e.g. diminished ULK1 (Ser^757^), S6K (Thr^389^) and 4E-BP1 (Thr^37/46^) phosphorylation, Fig. 4F). In shAMPK-transfected cultures, however, LC3B-II accumulation and ULK1 (Ser^317^), TSC2 (Ser^1387^) and raptor (Ser^792^) phosphorylation in response to trehalose were abrogated. However, despite AMPK knockdown and abrogation of distal AMPK signal transduction and AMPK-mTORC1 crosstalk, diminution of S6K (Thr^389^) and 4E-BP1 (Thr^37/46^) phosphorylation remained intact (Fig. 4F).

### GLUT8 is necessary for trehalose-induced AMPK signaling and autophagic flux, but is dispensable for trehalose-mediated mTORC1 suppression

**Figure 5:**
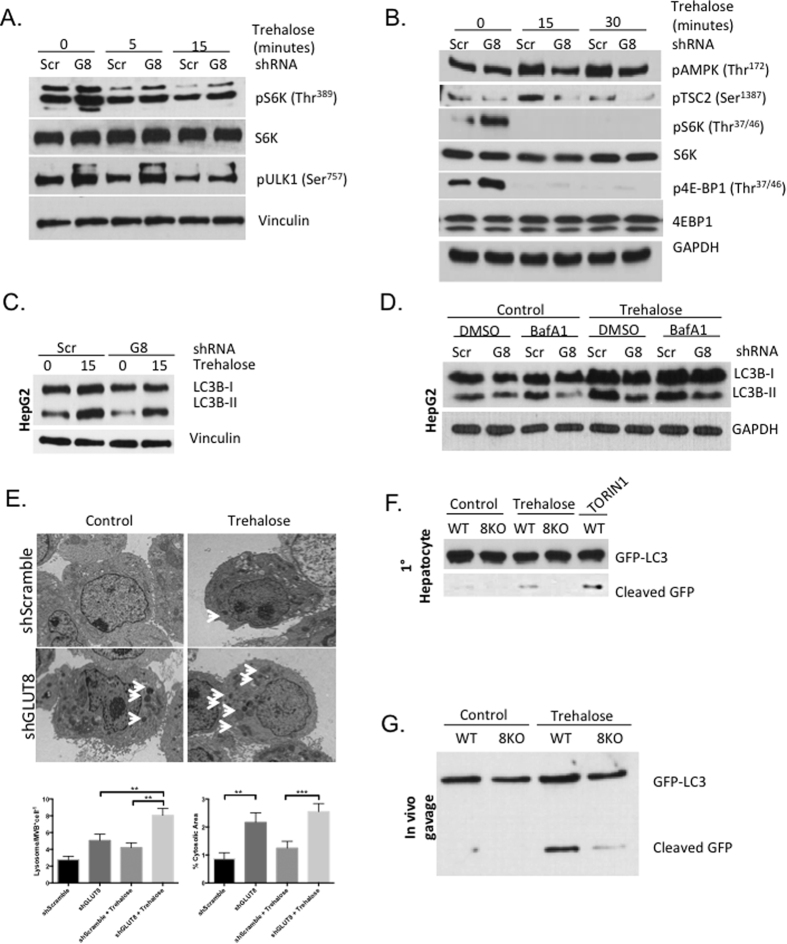
GLUT8 is required for trehalose-induced AMPK activation and autophagic flux. (**A**) GLUT8 is dispensable for trehalose-induced mTORC1 suppression. HepG2 cultures stably expressing scrambled or GLUT8-directed shRNA were treated with 100 mM trehalose (0–15 minutes), followed by phospho-specific mTORC1 target S6K (Thr^389^), ULK1 (Ser^757^) and 4E-BP1 (Thr^37^) immunoblotting. Vinculin was probed as a loading control. (**B**). GLUT8 is required for trehalose-induced AMPK activation. HepG2 cultures expressing scrambled or GLUT8-directed shRNA were treated with 100 mM trehalose (0–30 minutes) prior to phospho-specific immunoblotting against AMPK (Thr^172^) and AMPK target TSC2 (Ser^1387^) as well as mTORC1 targets 4E-BP1 (Thr^37^) and S6K (Thr^389^). (**C**) GLUT8 mediates trehalose-induced LC3B accumulation. HepG2 expressing scrambled shRNA or GLUT8-directed shRNA were treated with or without 100 mM trehalose (15 minutes, upper panels) 0–15 minutes prior to LC3B-specific immunoblotting. GAPDH is demonstrated as a loading control. (**D**). GLUT8 mediates trehalose-induced autophagic flux. HepG2 expressing scrambled shRNA or GLUT8-directed shRNA were treated with or without 100 mM trehalose (60 minutes) in the presence or absence of bafA1 prior to LC3B-specific immunoblotting. GAPDH is demonstrated as a loading control. (**E**) Electron micrographic analysis of shScramble and shGLUT8 HepG2 cultures treated with or without trehalose and bafA1 (1 hr). Arrowheads: multivesicular bodies and lysosome-like structures, identified by treatment-blinded microscopist. Lower bar graphs: lysosome/MVB-to-cytosol area ratios and quantity. ** and ***P < 0.01 and P < 0.001 between bracketed groups by one-way ANOVA. No other comparisons were significant. (**F**). GLUT8 is required for trehalose-induced GFP cleavage. Primary murine hepatocytes from GFP-LC3^WT^ and GFP-LC3^G8KO^ mice were treated with or without trehalose (100 mM) or TORIN1 30 minutes prior to lysis and GFP-specific immunoblotting. (**G**). GFP-LC3^WT^ and GFP-LC3^G8KO^ mice were subjected to orogastric gavage with oral saline or 6 g/kg trehalose. Crude liver lysates were subjected to GFP-specific immunoblotting.

Given the above findings that ULK and AMPK activation are required – but AMPK-mTORC1 crosstalk and mTORC1 suppression are dispensable – for trehalose-induced autophagy, we examined the role of GLUT8 in modulating trehalose-induced autophagy and signal transduction. shScramble and shGLUT8 cells were incubated in regular growth media or in 100 mM trehalose for 0–15 minutes prior to lysis and immunoblot analysis. Trehalose significantly reduced mTORC1 signaling in a time-dependent manner, with maximal inhibition observed 15-minutes post trehalose incubation (Fig 5A. Densitometric quantification data for Fig. 5 are catalogued in Supplemental Fig. 4). In contrast, AMPK phosphorylation was measured by immunoblotting in shScramble and shGLUT8 cells after treatment with or without 100 mM trehalose in regular growth media. Although mTORC1 substrate phosphorylation (4E-BP1 (Thr^37/46^) and S6K (Thr^389^)) was similarly suppressed in both trehalose-treated control and GLUT8-deficient cultures, AMPK (Thr^172^) and the AMPK substrate TSC2 (Ser^1387^) phosphorylation were stimulated only in trehalose-treated shScramble cultures. Indeed, trehalose-induced AMPK signaling was blunted in trehalose-treated GLUT8-deficient cells (Fig. 5B).

Evidence that 1) AMPK signaling is required for trehalose-induced hepatic autophagic flux independent of mTORC1 cross-talk, 2) GLUT8 is required for trehalose-induced AMPK activation and trehalose transport, and 3) intracellular trehalose is sufficient to induce cellular autophagy^3^ prompted us to test whether GLUT8 is indispensable for trehalose-induced autophagic flux. This hypothesis was first examined by immunoblot analysis in shScramble and shGLUT8 HepG2 cultures treated with or without trehalose (100 mM, 15′) in growth media. shScramble cells rapidly accumulated LC3B-II in response to trehalose, and this response was attenuated in parallel-treated shGLUT8 cultures (Fig. 5C). Autophagic flux was subsequently assayed in growth media- or trehalose-stimulated (100 mM, 1 hr) shScramble and shGLUT8 HepG2 cultures incubated with or without the vATPase inhibitor, bafA1 (100 nM, 1 hr). shScramble cultures exhibited robust LC3B-II accumulation in response to trehalose and bafA1 treatment. However, this LC3B-II response was blunted in identically treated GLUT8-deficient cultures (Fig. 5D). In contrast, consistent with prior reports^33^,^34^, trehalose-induced LC3B-II accumulation exhibited slower kinetics in N2A neuroblastoma cells (a cell type in which GLUT8 is not plasma-membrane localized). Moreover, trehalose-induced LC3B-II accumulation was not GLUT8-dependent in N2A neuroblastoma cells (not shown).

Consistent with biochemical data in hepatocytes, treatment-blinded analysis of electron micrographs obtained from bafA1-treated shScramble and shGLUT8 hepatocytes treated with or without trehalose (1 hr) revealed an overabundance of lysosomal and multivesicular bodies (Fig. 5E, arrowheads) in GLUT8-deficient hepatocytes. Treatment-blinded lysosome/MVB quantification and area measurement revealed that untreated GLUT8-deficient cells had significantly greater lysosome/MVB-to-cytosol area ratio without changes in number of lysosomes and MVBs. Trehalose treatment in wild-type cells induced trends toward increased lysosome and MVB quantity and % cytosol area ratio that did not reach statistical significance. In GLUT8-deficient cells, trehalose induced lysosome and MVB accumulation without altering the percentage of cytosolic area covered by these structures. Comparing trehalose-treated groups across genotypes, GLUT8-deficient cells had both higher lysosomal and MVB counts and a greater lysosome/MVB-to-cytosol area ratio when compared with wild-type controls (Fig 5E). Overall, electron micrographic analysis suggests enhanced trehalose-induced lysosome and MVB accumulation in GLUT8-deficient hepatocytes.

We then interrogated the role of GLUT8 in distinct, primary murine hepatocyte and *in vivo* GFP-LC3^WT^ and GFP-LC3^8KO^ models. We first quantified trehalose-induced GFP cleavage from GFP-LC3 fusion protein in primary GFP-LC3^WT^ and GFP-LC3^8KO^ hepatocytes incubated with or without trehalose (100 mM, 30 minutes) or with the mTORC1 inhibitor, Torin-1 (10 nM, 30 minutes) as a positive control. GFP-specific immunoblotting revealed that both Torin-1 and trehalose stimulated GFP cleavage in GFP-LC3^WT^ hepatocytes, whereas no GFP cleavage was detectable in GFP-LC3^G8KO^ hepatocytes (Fig. 5F).

The function of GLUT8 in hepatic autophagic flux *in vivo* was then assayed in non-fasting GFP-LC3^WT^ and GFP-LC3^G8KO^ subjected to orogastric saline or trehalose gavage (6 g/kg), followed by liver dissection, lysis and whole liver lysate immunoblotting. GFP-specific immunoblotting revealed that trehalose induced GFP cleavage from GFP-LC3 fusion protein *in vivo*, and this GFP cleavage response was attenuated in GFP-LC3^G8KO^ mice (Fig. 5G).

### GLUT8 mediates ameliorative effects of trehalose on fatty acid-induced hepatocyte triglyceride accumulation

**Figure 6:**
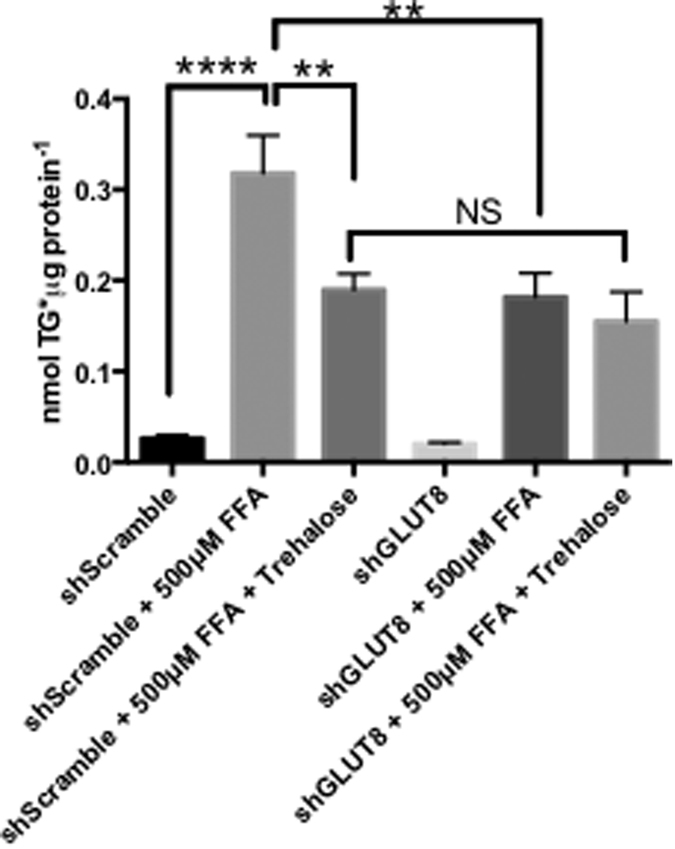
Trehalose prevents hepatic TG accumulation via a GLUT8-dependent mechanism. HepG2 cultures stably expressing control shRNA (shScramble) or GLUT8-directed shRNA (shGLUT8) were treated with 500 μM BSA-conjugated fatty acids with or without 100 mM trehalose (48hr) followed by TG determination. ** and ****P < 0.01 and P < 0.0001 between bracketed groups by one-way ANOVA with Sidak’s post-hoc correction for multiple comparisons. NS, P > 0.05 by one-way ANOVA.

Prior data indicated that hepatic GLUT8 ablation protects from fructose-induced hepatic steatosis^17^ and that trehalose-induced blockade of hexose transporters (primarily GLUT8 and GLUT2^17^) protects from fatty acid (FA)-induced steatosis^19^. We examined whether GLUT8 is required for trehalose-mediated protection from FA-induced steatosis by treating cultured shScramble or shGLUT8 hepatocytes in our *in vitro* triglyceride (TG) accumulation model. 500 μM FFA (48 hr) induced robust TG accumulation in shScramble hepatocytes, which was significantly mitigated by concomitant trehalose treatment (Fig. 6). GLUT8-deficient hepatocytes resisted FFA-induced TG accumulation, consistent with prior findings^17^,^19^. However, trehalose treatment in GLUT8-deficient hepatocytes treated with FA had no additive benefit with regard to triglyceride reduction (Fig. 6).

### Tret1 genetically complements autophagic and signaling defects in GLUT8-deficient hepatocytes

**Figure 7:**
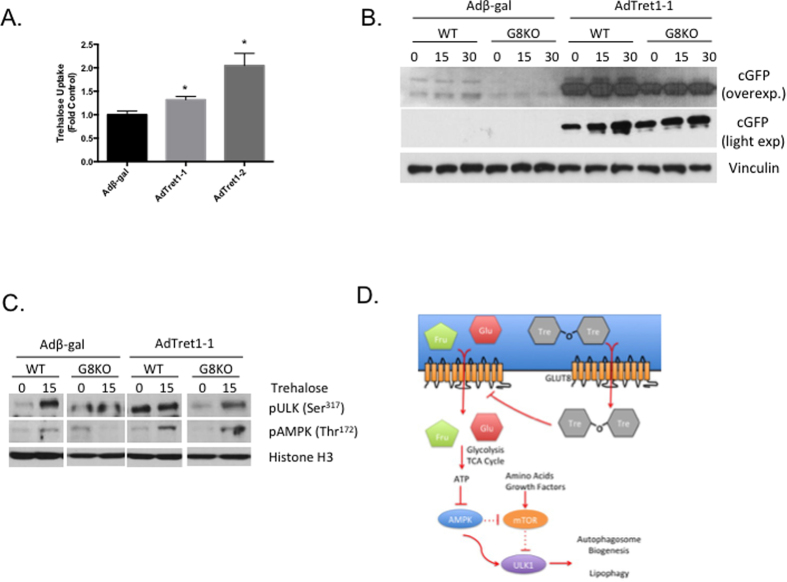
Drosophila Tret1 reconstitutes autophagic flux in GLUT8-deficient hepatocytes. (**A**). Radiolabeled trehalose uptake was quantified in HepG2 cultures transduced with adenovirus encoding β-galactosidase, Tret1-1 or Tret1-2 *P < 0.05, versus control by 2-tailed T-test. (**B**) dTret1-1 enhances hepatic GFP cleavage in GLUT8-deficient hepatocyte cultures. Primary GFP-LC3^WT^ and GFP-LC3^8KO^ hepatocytes were treated with trehalose (100 mM, 0–30 min) followed by GFP- and GAPDH-specific immunoblot analysis. (**C**) Tret1 reconstitutes trehalose-induced AMPK signal transduction in GLUT8-deficient primary hepatocytes. Primary GFP-LC3^WT^ and GFP-LC3^8KO^ hepatocytes were treated with trehalose (100 mM, 0–30 min) followed by phospho-ULK1 (Ser^317^), phospho-AMPK (Thr^172^) and histone H3-specific (loading control) immunoblot analysis D. Working model of GLUT8 function in trehalose-induced autophagy. GLUT8 mediates glucose, fructose and trehalose access to the hepatocyte cytoplasm to induce AMPK signaling. AMPK cross-talk with mTORC1, depicted by dashed lines, implies insufficiency of this arm of the pathway for autophagic flux.

GLUT8 is a Tret1 homolog required for hepatic autophagy via AMPK and ULK1 signal activation independent of AMPK-mTORC1 crosstalk. The hypothesis that Tret1 genetically complements GLUT8-mediated autophagic flux in response to trehalose was therefore examined in our hepatocyte culture models. We first tested the effect of Tret1-1 and Tret1-2 overexpression on hepatocyte trehalose uptake. Tret1-1 and Tret1-2 transfection both significantly upregulated acute trehalose uptake (5 minutes) in wild-type primary hepatocyte cultures (Fig. 7A). Primary hepatocytes from GFP-LC3^WT^ and GFP-LC3^8KO^ mice were then transfected with adenovirus encoding β-galactosidase (β-gal) or Tret1-1, stimulated in the presence or absence of 100 mM trehalose and subjected to immunoblot analysis. GFP-LC3^WT^ cultures responded to trehalose by increasing GFP-LC3 cleavage in a time-dependent manner, whereas GFP cleavage was attenuated in trehalose-treated GFP-LC3^8KO^ hepatocytes (Fig. 7B). In contrast, Tret1-1 transfection enhanced GFP cleavage in both basal and trehalose-treated GFP-LC3^WT^ and GFP-LC3^8KO^ hepatocyte cultures (Fig. 7B). Accordingly, Tret1-1 reconstituted trehalose-induced AMPK (Thr^172^) and ULK1 (Ser^317^) phosphorylation in GFP-LC3^8KO^ hepatocyte cultures after 15 minutes trehalose incubation (Fig. 7C). To quantify Tret1-1 overexpression in GFP-LC3^WT^ and GFP-LC3^8KO^ hepatocytes, primary cultures from each genotype were transfected with β-gal or HA-tagged Tret1-1. HA-tag immunoblot analysis revealed robust Tret1-1 overexpression in both GFP-LC3^8KO^ and GFP-LC3^WT^ hepatocytes (Supplementary Fig. 5A). Importantly, although we cannot definitively rule out whether differences in proteostasis *per se* underlie autophagic induction in GFP-LC3^8KO^ hepatocytes overexpressing Tret1-1, we observed that GFP cleavage was not associated with changes in ER stress marker gene expression (e.g. GRP78 or ATF4, Supplementary Fig. 5B) in either GFP-LC3^WT^ and GFP-LC3^8KO^ hepatocytes.

## Discussion

Known for its capacity to mitigate neurodegenerative disease and non-alcoholic fatty liver disease by inducing autophagic flux, the precise mechanisms by which trehalose stimulates autophagy have been elusive. Data from our group and others^3^,^10^,^19^ predicted trehalose action required efficient intracytoplasmic access not readily afforded by non-carrier-mediated mechanisms such as macropinocytosis or endocytosis^35^. Here, we demonstrate that trehalose accesses the hepatocyte interior at least in part via GLUT8, and that hepatic trehalose-induced autophagy depends in part upon a GLUT8-AMPK-ULK1 signaling cascade (Fig. 7D).

Kinetic data quantifying outside-in 2DG uptake are consistent with trehalose binding either outside the glucose binding pocket or within the glucose binding pocket in the inward open conformation^23^,^26^,^36^. Aligning with the latter possibility however, are 1) striking active-site identity between GLUT8 and Tret1 (Fig. 1B), trehalose receptor^19^ and trehalase^19^; 2) classical transporter-substrate modeling with glucose suggesting a thermodynamically plausible means by which a carbohydrate with a protected α-carbon (e.g. the α-α glucose-glucose linkage in trehalose) binds the inward open transporter conformation^37^ with corroborating trehalose-GLUT8 modeling in silico (Fig. 3); and 3) direct evidence that GLUT8 mediates trehalose transport (Fig. 2).

Intracytoplasmic glucose transport inhibition of both GLUT family and non-GLUT homolog hexose transporters is well documented. The HIV protease inhibitors^23^,^38^,^39^,^40^, gossypol^41^, ATP^42^, caffeine^43^, cytochalasin B^44^ and dehydroascorbic acid (DHA)^22^,^45^ inhibit GLUTs by binding the intracytoplasmic face of the transporter. Furthermore, at least in the instance of dehydroascorbic acid, access to the cell interior for DHA transport inhibition is mediated by GLUT8^22^ and GLUT1^45^. Data herein thus fit a described paradigm with regard to trehalose as both a transport substrate (or ligand) and concomitant inhibitor.

At present, data from our group and others indicate that trehalose inhibits multiple GLUT family members; GLUT8 is rate-limiting for trehalose transport into the cell; and intracellular trehalose synthesis is *sufficient* to induce autophagic flux^3^. Therefore, inward open conformation inhibition of glucose transport and consequent energetic deficits is consistent with previously proposed models of AMPK activation by trehalose^10^,^19^. Yet, there appears to be some selectivity with respect to the signaling link between GLUT8 and AMPK. It should be further acknowledged that the intracellular actions of trehalose may not be restricted to glucose transport inhibition. We cannot exclude the possibility, for instance, that the GLUT8 transporter acts concomitantly – or even primarily – either as a trehalose signaling receptor, or as an intracellular carbohydrate transporter (a concept previously postulated^11^,^46^,^47^) to activate AMPK and induce autophagy. This is supported by three pieces of evidence: 1) GLUT8 deficiency introduces an AMPK signal transduction defect, 2) trehalose-induced signal transduction via AMPK is reconstituted by Tret1 overexpression, 3) outside-in GLUT8-mediated trehalose transport capacity is relatively low when compared with *bona fide* substrate sugars (e.g. glucose and fructose) suggesting a role as an influx amplitude modulator (e.g. a “sensor”). Moreover, acknowledging *incomplete* abrogation of acute trehalose transport in contexts of GLUT8 deletion, GLUT8 knockdown and various GLUT inhibitors, there are likely to be additional means by which trehalose enters the hepatocyte. Regardless, GLUT8 seems to mediate an important signal and/or delivery mechanism in the trehalose-induced cascade. Analogously, GLUT2 is considered to be a signaling “transceptor” via binding and docking of signaling molecules such as L-pyruvate kinase to its large intracytoplasmic loop (homologous to the GLUT8 intracytoplasmic loop^48^,^49^). However, a “transceptor” hypothesis with respect to GLUT8 function in trehalose and other hepatic sugar-sensing action is speculative at this point.

Trehalose either directly or indirectly modulates pathways other than the AMPK-ULK pathway downstream of GLUT8. Concordant with several prior reports^50^, autophagy induced by trehalose was independent of mTORC1 activity. Strikingly, however, trehalose abrogated hepatic mTORC1 signaling through S6 kinase, ULK1 (Ser^757^) and 4E-BP1 (Fig. 5 and ref. 19), yet mTORC inhibition was insufficient to induce autophagic flux in HepG2 hepatocytes and in primary hepatocytes in concordance with prior reports^3^,^4^,^6^,^7^,^50^,^51^. Trehalose-induced autophagy may be cell-type specific with respect to mTORC1 blockade. Given also that trehalose is transported into the hepatocyte, and that it undergoes significant first-pass metabolism (less than 1% of orally dosed trehalose enters the peripheral circulation^19^, trehalose now has great potential to be a hepatoselective mTORC1 inhibitor apart from its autophagic effects. Although tissue-selective responses to trehalose require further characterization, the potential human therapeutic advantages of an mTORC inhibitor with this kinetic profile are numerous, given well-documented immunosuppressive side-effects of mTORC inhibitors (e.g. rapamycin) which escape hepatic first-pass metabolism.

Therapeutic implications for these findings may extend beyond metabolic control. Beyond autophagic induction in neurodegenerative disease and beyond mTORC1 inhibition in metabolic and growth derangements, multiple cancers overexpress GLUT8 and exhibit dependence upon this transporter for metabolic support of rapid growth, proliferation and invasion^13^,^52^. Although disaccharide transport is generally considered to be minimal or absent in mammalian tissue, recent findings suggesting that disaccharide moieties on cancer chemotherapeutics (e.g. bleomycin) enhance target-specific chemotherapeutic uptake^53^,^54^, resurrects the possibility that disaccharide transport into normal and abnormal mammalian tissue has implications for normal biology and for therapeutics across manifold pathophysiological states.

## Methods

### Mouse models

C57/B6 mice were obtained from the Jackson Laboratory (Bar Harbor, ME). GFP-LC3^WT^ and GFP-LC3^G8KO^ were obtained by crossing GLUT8-deficient mice^15^,^24^,^55^ with GFP-LC3 mice. Following interbreeding, mice were back-crossed on to a pure GFP-LC3^C57B/6^ background (total ≥4 times prior to experiment. All procedures were performed in accordance with the approved guidelines by the Animal Studies Committee at Washington University School of Medicine. All animal procedures were approved by the Washington University School of Medicine Animal Studies Committee.

### Cell Cultures

Methods used to generate the HEK 293 cell lines selectively overexpressing human GLUT8 with concomitant knockdown of endogenous GLUT1 via shRNA overexpression were recently described^19^,^23^. Absolute GLUT mRNA for the HEK GLUT8 overexpressing line and control line expressing GLUT1 shRNA are reported^19^,^23^.

HepG2 cultures were obtained from the American Type Culture Collection (Manassas, VA). Cultures were maintained in DMEM containing 4.5 g/L glucose and 5% FBS (e.g. “regular growth media”) through passage 20. To generate GLUT8-deficient HepG2 cell lines, HepG2 were transfected with lentivirus carrying the puromycin-resistance transgene and either scrambled shRNA-encoding vector (Catalog # SHC002V, Sigma-Aldrich, St. Louis, MO) or GLUT8-directed shRNA-encoding vector (Catalog # SHCLNV-NM_014580, Clone ID: NM_014580.3-630s21c1, Sigma-Aldrich, St. Louis, MO). All stably transfected cell lines were maintained under constant 2 μg/mL puromycin selection. Relative GLUT8 mRNA and protein knockdown is demonstrated in Supplemental Figures 1A,B.

Primary murine hepatocytes obtained from GFP-LC3^WT^ and GFP-LC3^G8KO^ were isolated as described^17^.

cDNA and adenoviral constructs used for transient transfections were obtained as follows: AMPKα-shRNA, tret1-1 and tret1-2 were purchased from Vector Biolabs (Malvern, PA). Wild-type ULK1, was purchased from Applied Biological Materials (Richmond, BC, Canada).

### Sequence Alignment and Phylogeny

Accession numbers for sequences aligned using Clustal Omega were Tret1-1 (NP_725068.1), Tret1-2 (AAM68715.1), GLUT1 (EDL30474.1), GLUT2 (NP_112474.2), GLUT3 (NP_008862.1), GLUT4 (NP_001033.1), GLUT5 (AAA52570.1), GLUT6 (NP_060055.2), GLUT7 (NP_997303.2), GLUT8 (NP_062361.1), GLUT9 (NP_064425.2), GLUT10 (NP_110404.1), GLUT11 (NP_110434.3), GLUT12 (NP_660159.1), HMIT (NP_443117.3). The following program-default parameters were used: Output format – Clustal without numbers; no input sequence dealignment; MBED-like clustering iteration and MBED-like clustering guide-tree; 0 combined guide-tree iterations; “default” maximum guide tree and “default” maximum HMM iterations were used; aligned order output.

### Fluorescence Microscopy and Multiphoton Imaging

HepG2 cells were plated on 35 mM glass-bottom dishes (MatTek, Ashland, MA). All images were collected using a Zeiss (Thornwood, NY) LSM510 META NLO multiphoton imaging system with 8000 nm excitation and a 40X lens. Preliminary experiments demonstrated closely related emission spectra when comparing cellular autofluorescence and tracer. To separate the tracer’s spectral signature we resorted to emission fingerprinting using tracer and unstained cells as reference. DMEM was replaced by 1 × PBS immediately before imaging to reduce background fluorescence. Emission spectra were acquired using beam splitter HFT-KP650, with an excitation wavelength of 800 nm.

### GC-MS Trehalose Quantification

Trehalose uptake quantification by gas chromatography and mass spectrometry was performed precisely as reported^19^. ^13^C_12_ trehalose (Omicron Biochemical, South Bend IN) was used as a sample extraction and derivatization internal standard. 5 nmol internal standard were added to the samples which were then extracted into 200 μl isopropanol:CH_3_CN:H_2_O. Samples were centrifuged and the supernatant was taken to dryness under nitrogen. Samples were derivatized using 100 ul N-Methyl-N-(Trimethysilyl) trifluoroacetamide (MSTFA): 10% pyridine in CH_3_CN (1:3) at room temperature overnight. Derivatized samples were analyzed using an Agilent 7890 A gas chromatograph interfaced to an Agilent 5975 C mass spectrometer. The GC column was a HP-5MS (30 m, 0.25 mm internal diameter, 0.25 um film coating, P.J. Cobert, St. Louis, MO). A linear temperature gradient was used. The initial temperature (80 °C) was held for 2 min and increased to 300 °C at 10 °C/min. The temperature was held at 300 °C for 2 min. Samples were run by electron ionization (EI) mode and the source temperature, electron energy and emission current were 200 °C, 70 eV and 300 μA, respectively. The injector and transfer line temperatures were 250 °C. The ions monitored were m/z 361 and 13-Trehalose at m/z 367.

### Radiolabeled Uptake Assays

GLUT8OE and control cell lines were grown in MEM supplemented with 10% FBS, 2 mM L-glutamine, 100 U/ml penicillin, and 100 μg/ml streptomycin (regular growth medium). [^14^C]-trehalose (American Radiolabeled Chemicals, St. Louis, MO) uptake was determined at 37 °C. To adhere the cells to tissue culture plates, dishes were first pretreated with 25 μg/ml polyethyleneimine (Fluka, catalog number P3143) in 150 mM NaCl for 20 min followed by the removal of the solution by vacuum. Cells were then plated at 2*10^5^ cells/ml MEM in regular growth medium in 12-well plates and incubated overnight at 37 °C. Following a 30-minute incubation at 37 °C in glucose-free HEPES, pH 7.3, 1 μCi/mL [^3^H]-2-deoxy-D-glucose (2DG) uptake was measured at 37 °C in glucose-free HEPES, pH 7.3 for 180 min at 37 °C. Cultures were washed extensively in cold phosphate-buffered saline and lysed in 1% triton X-100.buffer and 80% of each lysate was subjected to liquid scintillation counting (Ultima Gold, Thermo Fisher, Grand Island, NY). Protein concentration was determined in the remaining lysate and total protein per well was used to normalize total radiolabeled trehalose uptake in each well.

Primary hepatocytes and HepG2 cells were plated at 5*10^5^ cells per well in 12-well plates and maintained at 37 °C overnight in regular growth media until radiolabeled trehalose uptake was assayed. On the day of assay, cultures were incubated (15 minutes at 37 °C) in glucose-free HEPES, pH 7.3, in the presence or absence of unlabeled trehalose (50 mM), quercetin (50 μM, Sigma Aldrich, St. Louis, MO 21,22), lopinavir (20 μM, NIH AIDS Reagent Program, Germantown, PA^23^). Cultures were then incubated with 1 μCi/mL [^14^C]-trehalose (American Radiolabeled Chemicals, St. Louis, MO) in glucose-free HEPES, pH 7.3, at 37 °C (5 minutes for all inhibitor and knockdown studies, 180 minutes for the GLUT8OE experiments, consistent with prior trehalose transport kinetics^20^) prior to washing, lysis and analysis as described above.

### Modeling of GLUT8 and docking of trehalose to GLUT8 structures

GLUT8 structure was predicted using the I-TASSER webserver^56^,^57^,^58^. We selected the crystal structures of three close homologues through sequence alignment. The crystal structures of the human glucose transporter GLUT1 (4PYP), the bovine fructose transporter GLUT5 (4YB9), and the bacterial xylose transporter XylE (4GC0) in an open, inward-facing conformation were chosen as templates for the modeling. Trehalose was docked to the modeled structures of GLUT8 in the inward open conformation. We docked all three models of GLUT8 that were created using different template structures. The receptor (GLUT8) was prepared using MGL Tools 1.5.6, the ligand (trehalose) structure was energy minimized using ArgusLab 4.0.1 and prepared for docking using MGL Tools 1.5.6. Both molecules were docked in rigid receptor, flexible ligand mode using Autodock VINA^59^ with the search space covering the entire GLUT8 structure. The docking was performed using an exhaustiveness of 100. Figure 3 was generated using The PyMOL Molecular Graphics System, Version 1.8.07 Schrödinger, LLC.

### Immunoblotting

All immunoblot experiments depicted represent a minimum of three independent experiments with n = 2–3 per experiment. Immunoblotting was performed as described^16^. Primary antibodies were from Cell Signaling Technologies (Beverly, MA): AMPKα (#5831); phospho-AMPK (Thr^172^) (#2535); phospho-ULK1 (Ser^317^) (#2535); phospho-ULK1 (Ser^757^) (#6888); ULK1 (#8054); phospho-p70 S6K (Thr^389^) (#9234), p70 S6K (49D7, #2708); pTSC2 (Ser^1387^) (#5584); pRAPTOR (Ser^792^) (#2083); phospho-4E-BP1 (Thr^37/46^) (#2855); 4E-BP1 (#9644); HA-tag 6E2 (#2367); GFP (#2956); Vinculin (#13901); Actin (#4970); GAPDH (#5174); LC3B antiserum was obtained from Novus Biologicals (Littleton, CO) (#NB-100-2200).

For autophagic flux assays, primary hepatocytes and HepG2 cell lines were maintained in regular growth media prior to experiment. Cultures were then incubated in the presence or absence of dimethyl sulfoxide (DMSO) or 100 nM Bafilomycin A1 (Cayman Chemicals, Ann Arbor, MI) with or without 100 mM trehalose for 1 hour. Cultures were lysed and subjected to immunoblot analysis as above.

For *in vivo* autophagic flux assays, 8-week old GFP-LC3^WT^ and GFP-LC3^G8KO^ mice were gavaged with saline or with 6 g/kg trehalose dissolved in 0.9% NaCl. Mice were killed two hours after orogastric gavage, and livers were rapidly dissected and snap-frozen in liquid nitrogen prior to homogenization in ice-cold RIPA lysis buffer and immunoblot analysis.

For *in vivo* signaling assays, 8-week old wild-type mice were subjected to orogastric gavage with 6 g/kg trehalose dissolved in saline. Mice were killed 0.5 hours after gavage, and livers were rapidly dissected and snap frozen in liquid nitrogen prior to homogenization in ice-cold RIPA lysis buffer and immunoblot analysis. Control mice were gavaged with saline alone and killed 0.5 hours post-gavage.

### Transmission electron microscopy

Ultrastructural analysis was as previously performed^60^, with modifications. Cultured cells were fixed in 2% paraformaldehyde/2.5% glutaraldehyde (Polysciences Inc., Warrington, PA) in 100 mM sodium cacodylate buffer, pH 7.2 for 1 hr at room temperature. Samples were washed in cacodylate buffer and postfixed in 1% osmium tetroxide (Polysciences Inc.) for 1 hr. Samples were then rinsed extensively in ultrapure H_2_0 prior to en bloc staining with 1% aqueous uranyl acetate (Ted Pella Inc., Redding, CA) for 1 hr. Following several rinses in ultrapure H_2_0, samples were dehydrated in a graded series of ethanol and embedded in Eponate 12 resin (Ted Pella Inc.). Sections of 95 nm were cut with a Leica Ultracut UCT7 ultramicrotome (Leica Microsystems Inc., Bannockburn, IL), stained with uranyl acetate and lead citrate, and viewed on a JEOL 1200 EX transmission electron microscope (JEOL USA Inc., Peabody, MA) equipped with an AMT 8 megapixel digital camera and AMT Image Capture Engine V602 software (Advanced Microscopy Techniques, Woburn, MA).

A genotype- and treatment-blind expert electron microscopist calculated quantity and organelle-to-cytosolic area ratios by NIH imageJ 1.47 v analysis software. A lysosome/MVB was defined as any circumscribed, electron-dense intracytosolic structure containing variegated intraorganellar densities.

### *In Vitro* Triglyceride Accumulation Assays

For *in vitro* biochemical analysis, cultured hepatocytes were grown in 6-well plates and equilibrated 24 hr prior to treatment and assay as previously described^19^.

### Quantitative Real-Time RT-PCR (qPCR)

qPCR was performed with primer sequences and method precisely as described^17^. Primer sequences for GLUT family members are reported^19^,^23^.

### Statistics

Data were analyzed using GraphPad Prism version 6.0. P < 0.05 was defined as statistically significant after *post hoc* correction where multiple comparisons were made. Data shown are as mean ± SEM. Specific statistical tests applied are noted in the Figure Legends.

## Additional Information

**How to cite this article**: Mayer, A. L. *et al*. SLC2A8 (GLUT8) is a mammalian trehalose transporter required for trehalose-induced autophagy. *Sci. Rep.* **6**, 38586; doi: 10.1038/srep38586 (2016).

**Publisher's note:** Springer Nature remains neutral with regard to jurisdictional claims in published maps and institutional affiliations.

## Supplementary Material

Supplementary Figures
