# Supplementary material for: SLC2A8 (GLUT8) is a mammalian trehalose transporter required for trehalose-induced autophagy
Source: Sci Rep. 2016 Dec 6;6:38586. doi: 10.1038/srep38586 (PMC5138640; doi:10.1038/srep38586)
Supplement: Supplementary Figures [file srep38586-s1.pdf]

SUPPLEMENTARY INFORMATION

SLC2A8 (GLUT8) is a mammalian trehalose transporter required for trehalose-induced autophagy

Allyson L. Mayer[†a], Cassandra B. Higgins[†a], Monique R. Heitmeier[a], Thomas E. Kraft[a], Xia Qian[a], Jan R. Crowley[b], Krzysztof L. Hyrc[c,d], Wandy L. Beatty[e], Kevin E. Yarasheski[b], Paul W. Hruz[a] and Brian J. DeBosch*[,a,f]

[†]These authors contributed equally to this work.

*Corresponding author: 660 S. Euclid Ave., Box 8208, St. Louis, MO 63110. Email: debosch_b@kids.wustl.edu

[a]Department of Pediatrics, Washington University School of Medicine, 660 S. Euclid Ave., St. Louis, MO 63110, USA

[b]Department of Medicine, Washington University School of Medicine, 660 S. Euclid Ave., St. Louis, MO 63110, USA

[c]Center for the Investigation of Membrane Excitability Diseases, Washington University School of Medicine, 660 S. Euclid Ave., St. Louis, MO 63110, USA

[d]The Hope Center for Neurological Disorders, Alafi Neuroimaging Laboratory, Washington University School of Medicine, 660 S. Euclid Ave., St. Louis, MO 63110, USA

[e]Department of Molecular Microbiology, Washington University School of Medicine, 660 S. Euclid Ave., St. Louis, MO 63110, USA

[f]Department of Cell Biology & Physiology, Washington University School of Medicine, 660 S. Euclid Ave., St. Louis, MO 63110, USA

Supplemental Figure 1

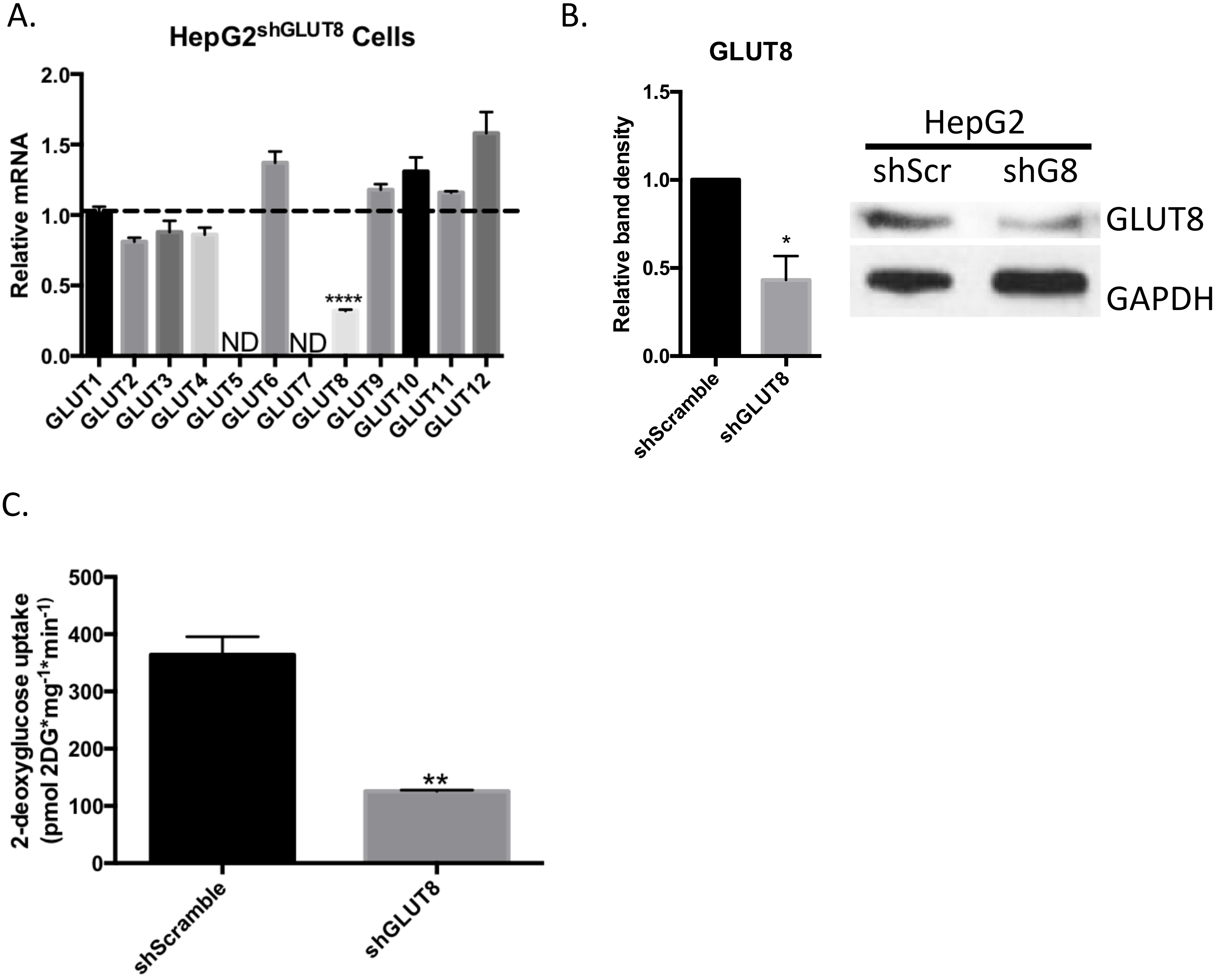

Supplemental Figure 1. Decreased GLUT8 mRNA, GLUT8 protein and glucose uptake in HepG2 cells expressing GLUT8-directed shRNA. A. GLUT family member expression in HepG2 cultures stably transfected with scrambled or GLUT8-specific shRNA. B. Immunoblot analysis and densitometric quantification of GLUT8 in HepG2 cells expressing scrambled or GLUT8-directed shRNA from n = 3 independent experiments. Each band is normalized internally by GAPDH band density. C. Radiolabeled 2-deoxyglucose uptake in HepG2 expressing scrambled or GLUT8-directed shRNA. *, $P<0.05$; **, $P < 0.01$ by 2-tail homoscedastic T-test.

Supplemental Figure 2

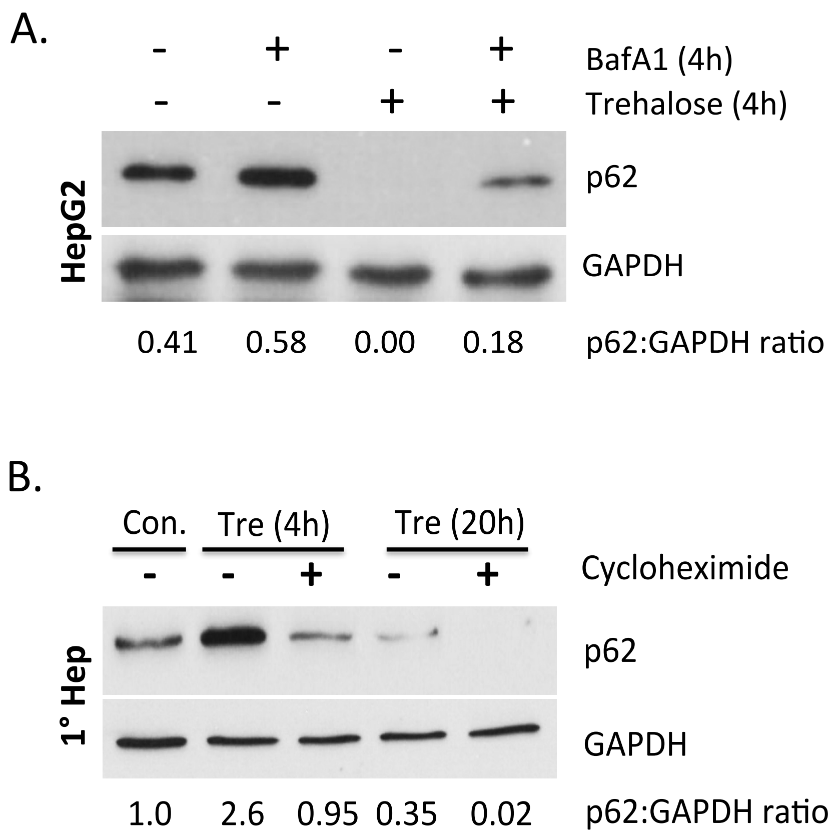

Supplemental Fig. 2. Trehalose induces p62 synthesis and degradation. A. p62 Immunoblot of lysates from HepG2 cells treated 4h with or without trehalose in the presence or absence of BafA1. B. p62 Immunoblot of lysates from primary hepatocytes treated with 100mM trehalose in the presence or absence of 10µM cycloheximide. p62:GAPDH density ratio is shown below each band.

Supplemental Figure 3

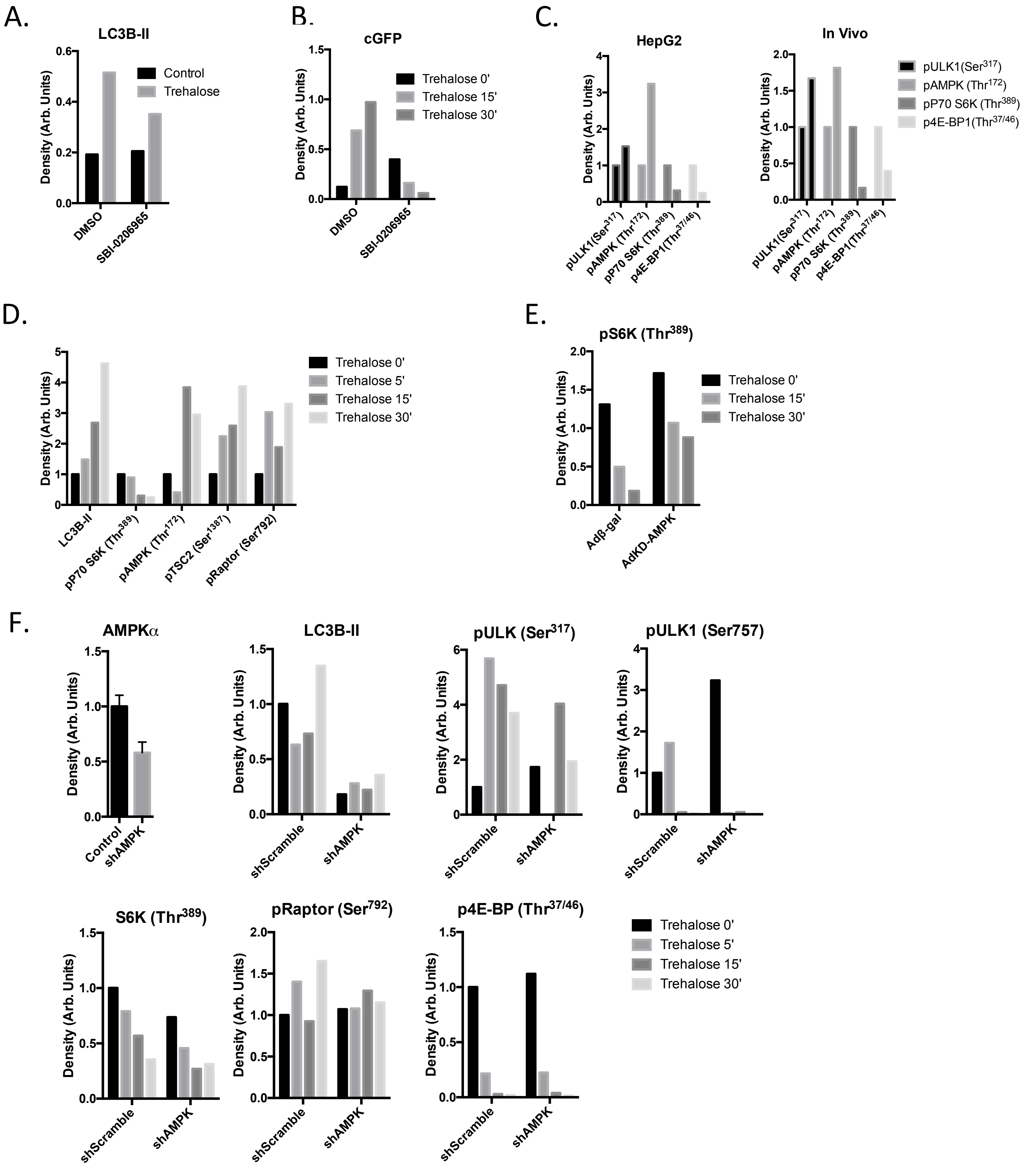

Supplemental Fig 3. AMPK and ULK1 activity – but not mTORC1 suppression - mediate trehalose-induced autophagy. A. Quantification of immunblot bands in Fig. 4A. B. Quantification of bands shown in Fig. 4B. C. Quantification of bands shown in Fig. 4C. D. Quantification of bands shown in Fig. 4D. E. Quantification of bands shown in Fig. 4E. F. Quantification of bands shown in Fig. 4F. All values are density ratios using housekeeping genes (e.g. actin, GAPDH, vinculin) as internal loading controls.

Supplemental Figure 4

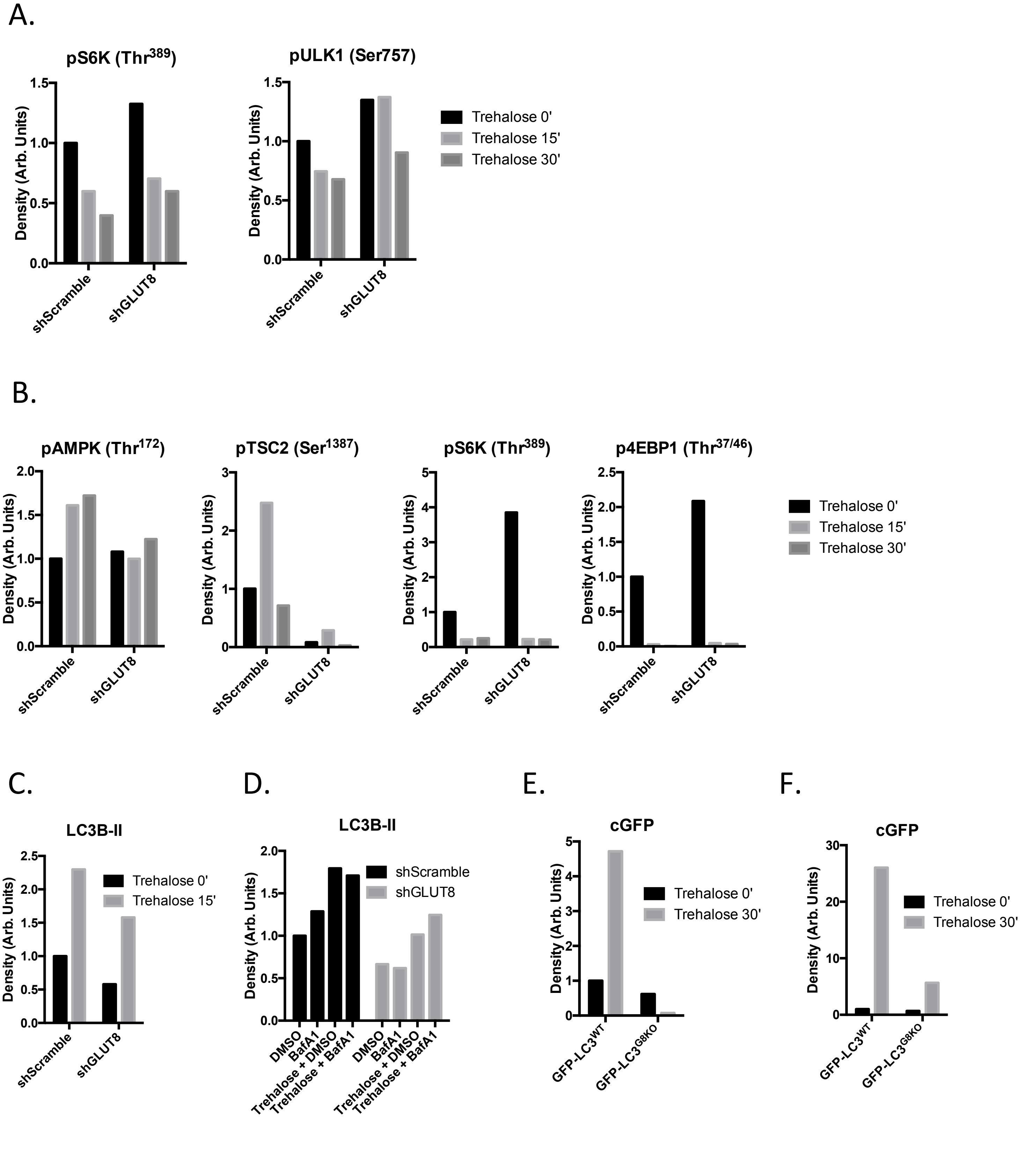

Supplemental Fig 4. GLUT8 is required for trehalose-induced AMPK activation and autophagic flux A. Quantification of immunblot bands in Fig. 5A. B. Quantification of bands shown in Fig. 5B. C. Quantification of bands shown in Fig. 5C. D. Quantification of bands shown in Fig. 5D. E. Quantification of bands shown in Fig. 5F. F. Quantification of bands shown in Fig. 5G. All values are density ratios using housekeeping genes (e.g. actin, GAPDH, vinculin) as internal loading controls.

Supplemental Figure 5

A.

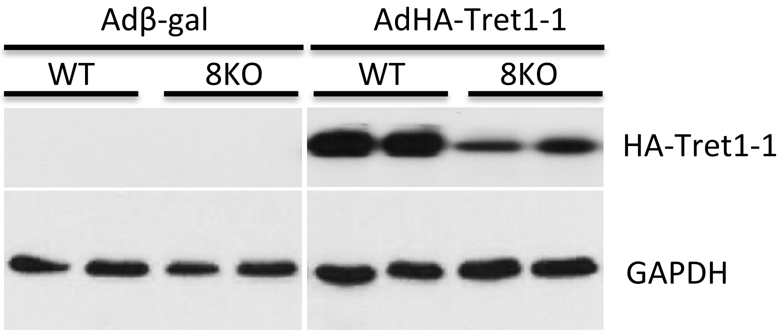

B.

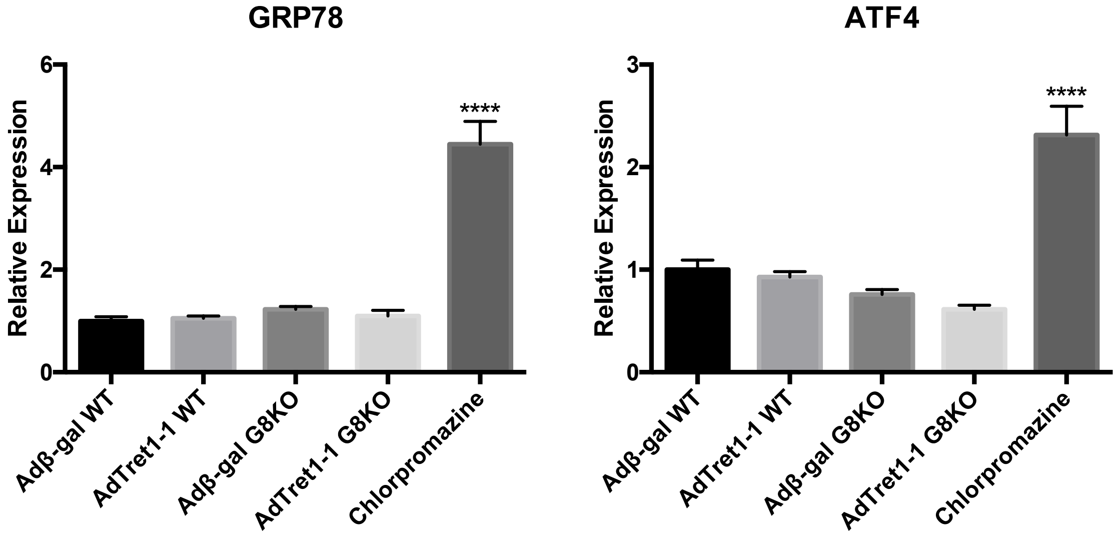

Supplemental Fig 5. A. Immunoblot analysis of Tret1-1 overexpression in primary WT and 8KO hepatocytes. B. Adenoviral Tret1 overexpression does not induce GRP78 or ATF4 expression. Primary hepatocytes were transfected with adenovirus encoding β-galactosidase or Tret1-1 or treated with chlorpromazine for 48hr prior to qRT-PCR for GRP78 or ATF4. N = 3 independent cultures per treatment. Tret1-1-transfected groups are not statistically different from the others by one-way ANOVA. ****, P< 0.0001 versus β-gal transfected by one-way ANOVA for n = 6 independent measurements per group.
